# Red, white, and processed meat consumption related to inflammatory and metabolic biomarkers among overweight and obese women

**DOI:** 10.3389/fnut.2022.1015566

**Published:** 2022-11-10

**Authors:** Farideh Shiraseb, Dorsa Hosseininasab, Atieh Mirzababaei, Reza Bagheri, Alexei Wong, Katsuhiko Suzuki, Khadijeh Mirzaei

**Affiliations:** ^1^Department of Community Nutrition, School of Nutritional Sciences and Dietetics, Tehran University of Medical Sciences (TUMS), Tehran, Iran; ^2^Department of Nutrition, Science and Research Branch, Islamic Azad University, Tehran, Iran; ^3^Department of Exercise Physiology, University of Isfahan, Isfahan, Iran; ^4^Department of Health and Human Performance, Marymount University, Arlington, VA, United States; ^5^Faculty of Sports Sciences, Waseda University, Tokorozawa, Japan

**Keywords:** red meat, white meat, inflammatory markers, processed meat, obesity, women

## Abstract

**Background:**

Considering that a high meat intake is directly associated with obesity, it is critical to address the relationship between consuming different types of meat with inflammation and metabolism in overweight and obese cohorts. Thus, we evaluated the association between red, white, and processed meat consumption with inflammatory and metabolic biomarkers in overweight and obese women.

**Methods:**

The current cross-sectional study was conducted on 391 overweight and obese Iranian women. Dietary intake was obtained from a food frequency questionnaire (FFQ) with 147 items. The anthropometric measurements, serum lipid profile, and inflammatory markers were measured by standard protocols. All associations were assessed utilizing one-way analysis of variance (ANOVA), analysis of covariance (ANCOVA), and linear regression models.

**Results:**

In the adjusted model, it was established that higher intake of processed meat had a significant positive association with leptin levels (β: 0.900, 95% CI: 0.031;1.233, *p* = 0.015). Moreover, after considering the confounders, a significant positive association between processed meat and macrophage inflammatory protein (MCP-1) levels was observed (β: 0.304, 95% CI:0.100;1.596, *p* = 0.025). Positive significant associations between high-sensitivity C-reactive protein (hs-CRP) (β:0.020, 95% CI:0.000;0.050, *P* = 0.014) and plasminogen activator inhibitor 1 (PAI-1) (β:0.263, 95% CI:0.112;0.345, *p* = 0.053) and MCP-1 (β:0.490, 95% CI: 0.175;1.464, *p* = 0.071) levels with red meat were also shown; while there was a significant negative association between red meat and the homeostasis model assessment of insulin resistance (HOMA-IR) (β: −0.016, 95% CI: −0.022, −0.001, *p* = 0.033). Furthermore, a significant negative association were established following confounding adjustment between Galectin-3 (Gal-3) (β: −0.110, 95% CI: −0.271;0.000, *p* = 0.044), MCP-1 (β: −1.933, 95% CI: −3.721;0.192, *p* = 0.022) and Homeostatic Model Assessment for Insulin Resistance (HOMA-IR) (β: −0.011, 95% CI: −0.020,0.000, *p* = 0.070) levels with high adherence of white meat intake. In contrast, a significant marginally positive association between PAI-1 levels and high adherence to white meat intake (β: −0.340, 95% CI: −0.751;0.050, *p* = 0.070) has been shown.

**Conclusions:**

Higher red and processed meat consumption were positively associated with inflammatory and metabolic markers in overweight and obese women. In contrast, negative relationships between high adherence to white meat and various inflammatory and metabolic parameters were established. Further studies are needed to confirm the causality of these associations and potential mediating pathways.

## Introduction

Global obesity rates are rising (1). Currently, more than 26% of Iranian adults are obese, however, Iranian women are more affected than males (57 vs. 22%) (2). Obesity is defined as a genetically based chronic multifactorial condition that is brought on by the buildup of extra fat tissue (3). Numerous serious comorbidities are caused by it, including insulin resistance, hypertension, diabetes mellitus, and low-grade inflammation (1, 4–6). Excess adipose tissue produces and secretes an increasing number of inflammatory mediators into the systemic circulation, enhancing the inflammatory profile (7, 8). Among these are acute-phase proteins, including plasminogen activator inhibitor 1 (PAI-1), and classic peptide mediators of inflammation such as interleukin 1 (IL-1) (9, 10), macrophage inflammatory protein (MCP-1) (11), transforming growth factor (TGF-b) (12), and high-sensitivity C-reactive protein (hs-CRP) (13). Moreover, increasing abdominal fat mass is associated with insulin resistance as one inflammation indicator (14) and ablation of Galectin-3 (Gal-3), which hastens lipid-induced atherogenesis and plays an essential role in cell-cell adhesion, cell-matrix interactions, macrophage activation, metastasis, and apoptosis (15, 16).

Various factors are directly related to causing obesity and as a result, causing inflammation or changes in inflammatory levels, but undoubtedly one of the most important factors is food intake, which is, directly and indirectly, effective in changing inflammatory levels (17, 18). The food sources received from the main food groups, especially protein intake from different sources, are effective in causing obesity and consequently inflammation, or directly in the occurrence of inflammation (19–21). One of the food items that have conflicting and challenging results in studies on inflammation and metabolic diseases is getting protein from different food sources, especially red and processed meat (22–24). Research to date indicates that a high meat intake is directly associated with obesity. Indeed, inflammation and insulin resistance related to excess adipose tissue have been proposed to explain the documented link between red meat consumption and metabolic disorders in obese cohorts (22–24). This relationship may be explained by the negative effects of saturated fat, animal protein, and red meat's high iron content, mainly heme iron, saturated fatty acid (SFA) that has been linked to increased adiposity, inflammation, and insulin resistance (IR) (25–27). On the other hand, several studies have shown that fish and its components favor inflammatory markers (28–30). For instance, eating white meat improves interleukin 6 (IL-6) synthesis, affecting hs-CRP development in the liver (21). Despite these findings, investigations evaluating the relationship between consuming different types of meat and inflammatory and metabolic markers in overweight and obese cohorts are scarce. Addressing these relationships in overweight and obese women is particularly important, as they are more likely to develop inflammatory and metabolic abnormalities than their male counterparts (31, 32).

According to an explanation and despite the existing controversies regarding the relationship between the consumption of red and processed meat with inflammatory factors needed. In addition, this issue has not been investigated sufficiently in Iran, especially in obese and overweight women, so with a view more comprehensively, the intake of red, processed, and white meat was examined in the present study. Therefore, we evaluated the relationship between red, white, and processed meat consumption with inflammatory and metabolic biomarkers in overweight and obese women.

## Materials and methods

### Participants

In this cross-sectional study recruited, a total of 391 healthy overweight (BMI = 25–29.9), and obese (BMI ≥ 30) women, aged 18–56 years old that had been referred to health centers in Tehran. The exclusion criteria were as follows: smoking; chronic disease histories such as the history of hypertension, cardiovascular diseases, diabetes mellitus, impaired renal and liver function inflammatory diseases, cancer, thyroid disease, regular use of medicine (including oral contraceptive pill), alcohol use, supplement consumption (vitamins and minerals and both) pregnancy, lactation period, and menopause, people who had been on an arbitrary special diet plan and anyone whose body weight had changed noticeably during the previous year, participated in sports, if their total calorie consumption did not fall between 800 and 4,200 (17,556–3,344 kJ) (33), and individuals with weight fluctuations in the past year were also excluded from the study. Before participating in the study, each participant signed a written informed consent form. The study was approved by the Ethical Committee of the Tehran University of Medical Sciences (TUMS) and performed according to the ethical standards of the Declaration of Helsinki (IR.TUMS.VCR.REC.1395.1597).

### Study design

In this descriptive cross-sectional study, sampling and data collection were completed in 2018. Multi-stage random cluster sampling was performed among health centers affiliated with the TUMS to select certain regions from among all the regions of the city; 20 clusters were selected. Two visits were conducted: in the first visit, a demographic questionnaire, food frequency questionnaire (FFQ), blood pressure as well as anthropometric and body composition measurements were performed. In the second visit, blood samples were taken from individuals. All measurements were performed in the Nutrition and Biochemistry Laboratory of the School of Nutritional and Dietetics at TUMS.

### Dietary assessments

Dietitians assessed dietary intake using a validated 147-item semi-quantitative food frequency questionnaire (FFQ) by face-to-face interview (34, 35). Individuals reported the frequency of each food item consumed in the past year, which was consequently converted to grams per day using household measures (36). Energy and dietary nutrients were calculated using the Iranian Food Composition Table and NUTRITIONIST IV (version 7.0; N-Squared Computing, Salem, OR). The current study is based on the consumption of three different types of meat extracted from the FFQ as gram/day: (1) the red meat category was defined as the sum of red (beef, lamb, sheep) and organ meats (beef liver, kidney, tongue, and heart); (2) white meat consisted of fish and poultry, such as chicken and turkey; and (3) processed meats included sausages, hamburger, Kalbas, Mortadella, and canned fish. All kinds of meat intake were adjusted by energy intake by residual method.

### Anthropometric and body composition assessment

The anthropometrics and body composition examination were conducted between 8 and 9 a.m., following a 12-h overnight fast. Participants also abstained from unusual physical activity for 72 h prior to the assessments. Weight was measured using a digital scale (Seca, Hamburg, Germany) in light clothing and without shoes with a precision near 0.1 kg, and stature was measured *via* a stadiometer with an accuracy close to 0.1 cm. Hip circumference (HC) and waist circumference (WC) were measured separately in the smallest and largest girth, respectively, with accuracy nearest to 0.1 cm. A multi-frequency bioelectrical impedance analyzer (BIA) called the InBody 770 Scanner was used to measure body composition (Inbody Co., Seoul, Korea). Using electrical impulses from the hands and feet, this device measures the resistance of bodily tissues. Participants were instructed to thoroughly urinate (void) and refrain from drinking water 30 min before the test. Participants were instructed to remove any metal objects, including tools, jewelry, coats, jackets, and shoes, following the manufacturer's instructions (25). They were also requested to remove their socks before the device was placed on them, to boost accuracy. Our BIA in our lab has a test-retest reliability of r = 0.98.

BMI, skeletal muscle mass (SMM), fat-free mass (FFM), body fat mass (BFM), bone mass content (BMC), soft lean mass (SLM), visceral fat level (cm), trunk fat, waist to hip ratio (WHR), fat mass index (FMI), fat-free mass index (FFMI) as body composition components were established with a BIA.

### Biochemical assessment

Blood samples were taken after a 10–12 h fast, and serum was stored at −80 °C. All tests were analyzed according to the manufacturer's guidelines. The glucose oxidase method was utilized for fasting blood sugar (FBS) appraisal, and the affront level was measured by an enzyme-linked immunosorbent measure (ELISA) unit (Human affront ELISA unit, DRG Pharmaceuticals, GmbH, Germany). Triglyceride (TG), total cholesterol (Chole), Low-density lipoprotein cholesterol (LDL-c), and High-density lipoprotein cholesterol (HDL-c) were measured by related packs (Pars Azemun, Iran). Serum glutamic pyruvic transaminase (SGPT) and serum glutamic-oxaloacetic transaminase (SGOT) were evaluated utilizing the Universal League of Clinical Chemistry and Research facility Medication standardization. The HOMA-IR was assessed as the item of fasting glucose and affront level isolated by 22.5 with a molar unit (mmol/L) (37). The Enzyme-Linked Immunosorbent Assays (ELISA) technique was used to evaluate the levels of inflammatory biomarkers (such as hs-CRP and IL-1b), Gal-3. (R&D Systems, Minneapolis, MN), MCP-1 (Zell Bio GmbH, ULM, Germany), TGF-β (HUMAN TGF-BETA 1 Quantikine ELIZA kit R&D System-USA), PA-I (Human PAI-1^*^96 T ELIZA kit Crystal Company) and serum leptin concentrations (Mediagnost, Reutlingen, Germany). The inter-assay and intra-assay variability for all tests were <12 and 10%, respectively.

### Blood pressure

Systolic and diastolic blood pressure (SBP, DBP) were assessed after 15 min of rest using an automatic sphygmomanometer (OMRON, Germany). Three measurements at 1-min intervals were taken and averaged.

### Demographic variables and physical activity

A demographic questionnaire was utilized to assess quality characteristics such as education, employment, marital status, economic levels, and family history of obesity. The validated International Physical Activity Questionnaire (IPAQ) was converted to Minutes per week using Metabolic Equivalents (MET-min/week) to determine physical activity criteria (38).

### Statistical analysis

The sample size was computed according to the following formula: n = [(Z 1-α + Z1-β) × (√1- r^2^)/r)^2^] which r = 0.21, β = 0.90 and α = 0.05. The evaluation of the histogram curve and the Kolmogorov-Smirnov test was used to ensure that the data were distributed normally (*p* > 0.05). In addition according to the central limit theorem, all dependent variables are taken into account using the normal distribution (39, 40). The mean and standard deviation were used to describe quantitative variables, whereas number and percent (%) were used to represent categorical variables. One-way analysis of variance (ANOVA) was validated for assessing the mean difference of quantitative variables across red, processed, and white meat' medians. Relationships between inflammatory indicators were investigated, and analysis of covariance (ANCOVA) was employed to control the effect of confounders in 2 models. The Chi-square (χ^2^) test was used to examine categorical variables. Three models used linear regression to control for confounders and covariates points and validated associations between red, processed, and white meat consumption and inflammatory markers. This analysis was present by beta (β) and 95% Confidence Interval (CI) and goodness of fit (GOF) of r squared (R^2^). All linear regression analysis's assumptions and concerns, including normality, the normality of residual error, linearity, homoscedasticity, and collinearity, were evaluated. IBM SPSS version 26.0 was used to conduct statistical analyses (SPSS, Chicago, IL, USA). The significance level was set at 2-sided *P* < 0.05 and *P* = 0.05–0.07, considered marginally significant.

## Results

### Study population characteristics

The present study was conducted on 391 obese and overweight Iranian women, of which 27% were single, 46.8% had a college education, and 27.3% had good economic status. The means and standard deviation (SD) of age, weight, BMI and WHR, FFM, and BFM of individuals were 36.681 (9.150) years, 80.320 (11.065) kg, 31.011 (3.920) kg/m^2^, 1.162 (4.575), 46.523 (5.690) kg and 34.804 (8.800) kg, respectively. Moreover, the mean intake of red meat was 40.161 (19.660) gr/day, while white and processed meat had mean intakes of 59.300 (40.421) gr/day and17.896 (14.325) gr, respectively.

### Population characteristics among medians of red, white, and processed meat consumption

General characteristics of participants, such as body composition, biochemical assessment, and others among lower vs. higher than the median of processed meat, red meat, and white meat intake, are presented in Table 1. *P*-values for all variables were reported before the adjustment in the crude model by ANOVA, and after adjustment with potential confounders, including age, BMI, physical activity, and energy intake (Table 1).

**Table 1:** Study participant characteristics between medians of processed meat, red meat, and white meat (g/d) in 391 obese and overweight women.

**Variables**	**Processed meat median**		***P*** **-value**	***P*** **-value^†^**	**Red meat median**		***P*** **-value**	***P*** **- value^†^**	**White meat median**		***P*** **-value**	***P*** **-value^†^**
	**Low (<19.82)**	**High** **(>19.83)**			**Low (<36.46)**	**High** **(>36.47)**			**Low (<49.56)**	**High** **(>49.57)**		
Age (years)	34.401 ± 0.856	36.150 ± 0.874	0.094	0.841	36.930 ± 0.774	35.400 ± 0.781	0.102	0.181	36.400 ± 0.812	36.001 ± 0.734	0.255	0.726
Physical activity (MET-minutes/week)	1,081.961 ± 149.235	896.100 ± 153.414	0.681	0.396	1,000.751 ± 191.942	1,361.091 ± 195.476	0.212	0.200	1,060.001 ± 201.422	1,273.274 ± 181.101	0.725	0.440
**Anthropometric variables**												
Weight (kg)	80.337 ± 0.692	78.035 ± 0.682	0.405	**0.024**	79.321 ± 0.677	78.947 ± 0.716	0.101	0.706	78.958 ± 0.688	79.322 ± 0.685	0.752	0.706
Height (cm)	162.062 ± 0.645	160.255 ± 0.624	0.650	**0.050**	161.331 ± 0.616	160.997 ± 0.643	0.851	0.701	161.041 ± 0.624	161.307 ± 0.626	0.186	0.770
HC (cm)	105.361 ± 0.224	105.085 ± 0.215	0.355	0.397	105.120 ± 0.216	105.271 ± 0.220	0.094	0.648	104.988 ± 0.214	105.400 ± 0.210	0.730	0.160
WC (cm)	98.102 ± 0.520	97.362 ± 0.511	0.590	0.321	99.325 ± 0.374	98.182 ± 0.372	0.112	**0.034**	97.950 ± 0.504	97.504 ± 0.517	0.295	0.535
NC (cm)	38.265 ± 0.915	36.877 ± 0.908	0.651	0.297	37.200 ± 0.875	37.978 ± 0.926	0.752	0.545	36.855 ± 0.882	38.292 ± 0.881	0.282	0.252
BMI (kg/m^2^)	30.50 ± 0.387	30.77 ± 0.398	0.395	0.634	30.868 ± 0.344	30.272 ± 0.355	0.286	0.242	30.100 ± 0.351	30.969 ± 0.326	0.112	**0.072**
**Body composition**												
WHR	0.931 ± 0.000	0.924 ± 0.000	0.161	0.542	0.934 ± 0.000	0.921 ± 0.000	0.302	0.215	0.930 ± 0.000	0.920 ± 0.000	0.314	0.264
WHtR	0.601 ± 0.000	0.601 ± 0.000	0.800	0.437	0.608 ± 0.000	0.605 ± 0.000	0.171	0.282	0.608 ± 0.000	0.605 ± 0.000	0.437	0.341
BFM (%)	40.687 ± 0.385	41.342 ± 0.377	0.530	0.611	40.824 ± 0.367	41.202 ± 0.386	**0.071**	0.487	41.278 ± 0.372	40.732 ± 0.376	0.664	0.315
FFM (kg)	47.325 ± 0.566	45.567 ± 0.555	0.154	**0.030**	46.644 ± 0.547	46.188 ± 0.572	0.294	0.567	46.214 ± 0.552	46.637 ± 0.550	0.861	0.603
SMM (kg)	25.954 ± 0.334	25.033 ± 0.324	0.151	**0.054**	25.600 ± 0.314	25.350 ± 0.331	0.620	0.590	25.314 ± 0.324	25.657 ± 0.326	0.843	0.460
SLM (kg)	44.615 ± 0.521	42.951 ± 0.511	0.152	**0.022**	43.977 ± 0.502	43.534 ± 0.532	0.336	0.550	43.584 ± 0.513	43.952 ± 0.517	0.691	0.612
FFMI	17.986 ± 0.112	17.722 ± 0.112	0.140	0.110	17.866 ± 0.101	17.821 ± 0.112	0.342	0.791	17.761 ± 0.100	17.932 ± 0.100	0.221	0.262
FMI	12.544 ± 0.110	12.834 ± 0.110	0.966	**0.062**	12.631 ± 0.100	12.734 ± 0.111	0.110	0.500	12.751 ± 0.114	12.612 ± 0.114	0.204	0.387
VFL (cm^2^)	15.197 ± 0.201	15.191 ± 0.201	0.767	0.994	15.163 ± 0.191	15.223 ± 0.200	0.241	0.837	15.334 ± 0.190	15.0415 ± 0.19	0.874	0.301
Trunk fat (%)	303.765 ± 2.340	309.651 ± 2.304	0.794	**0.041**	306.467 ± 2.242	306.674 ± 2.372	0.154	0.941	308.303 ± 2.272	304.793 ± 2.272	0.160	0.281
BMC (kg)	2.641 ± 0.024	2.584 ± 0.031	0.260	0.125	2.661 ± 0.030	2.642 ± 0.041	0.241	0.781	2.633 ± 0.031	2.671 ± 0.031	0.642	0.491
**Biochemical components**												
TG (mg/dl)	112.662 ± 7.061	120.932 ± 7.502	0.560	0.441	123.361 ± 5.860	104.542 ± 5.900	0.64	**0.023**	110.231 ± 6.870	117.292 ± 5.823	0.587	0.903
TC (mg/dl)	176.521 ± 3.501	176.911 ± 3.720	0.584	0.943	182.141 ± 3.450	182.231 ± 3.472	0.971	0.980	180.872 ± 3.943	181.597 ± 3.348	**0.030**	0.701
HDL (mg/dl)	47.564 ± 1.114	45.932 ± 1.174	0.253	0.332	46.571 ± 1.092	46.955 ± 1.102	0.511	0.802	44.604 ± 1.220	48.111 ± 1.032	**0.070**	0.152
LDL (mg/dl)	96.036 ± 2.495	96.221 ± 2.652	0.481	0.962	92.981 ± 2.342	93.592 ± 2.351	0.800	0.851	91.301 ± 2.612	94.582 ± 2.211	0.4264	0.57
FBS (mg/dl)	85.682 ± 1.055	87.832 ± 1.112	0.662	0.172	87.362 ± 0.932	86.964 ± 0.930	0.222	0.761	86.864 ± 1.005	87.241 ± 0.852	**0.041**	0.272
Insulin (mIU/ ml)	1.200 ± 0.022	1.194 ± 0.022	0.500	0.804	1.236 ± 0.025	1.183 ± 0.025	0.191	0.131	1.200 ± 0.021	1.191 ± 0.022	0.145	0.731
GOT (μKat/L)	17.951 ± 0.781	16.442 ± 0.8320	0.320	0.201	18.242 ± 0.771	17.421 ± 0.783	0.602	0.4611	17.971 ± 0.672	17.134 ± 0.671	0.362	0.590
GPT (μKat/L)	18.662 ± 1.421	17.881 ± 1.510	0.752	0.722	19.970 ± 1.375	17.765 ± 1.381	0.411	0.260	18.862 ± 1.401	17.861 ± 1.190	0.562	0.922
SBP (mmHg)	112.845 ± 1.532	109.340 ± 1.090	**<0.001**	0.130	110.892 ± 1.372	111.9303 ± 1.38	0.952	0.596	111.822 ± 1.586	110.660 ± 1.341	0.531	0.213
DBP (mmHg)	78.310 ± 1.090	77.380 ± 1.150	0.150	0.574	77.431 ± 0.975	77.485 ± 0.972	0.481	0.960	78.440 ± 1.091	76.9110 ± 0.92	0.510	0.452
**Categorical variables**												
**Economic status**												
Low level	55 (77.5)	16 (22.5)	**0.043**	**0.011**	58 (65.9)	30 (34.1)	**0.001**	**0.003**	50 (56.8)	38 (43.2)	**0.012**	**0.022**
Moderate level	105 (66.5)	33.5 (53)			86 (47.3)	96 (52.7)			92 (50.5)	90 (49.5)		
High level	55 (59.13)	38 (40.9)			42 (39.3)	65 (60.7)			39 (36.4)	68 (63.6)		
**Education level**												
Illiterate	1 (50)	1 (50)	0.941	0.782	2 (50)	2 (50)	**0.009**	**0.002**	2 (50)	2 (50)	**0.003**	**0.001**
Under diploma	17 (48.6)	18 (51.4)			34 (69.4)	15 (30.6)			30 (61.2)	19 (38.8)		
Diploma	43 (47.8)	47 (52.2)			78 (51.7)	73 (48.3)			83 (55)	68 (45)		
Master and higher	54 (51.9)	50 (48.1)			80 (43.2)	105 (56.8)			70 (37.8)	115 (62.2)		
**Marital status**											**0.05**	0.08
Single	25 (50)	25 (50)	0.971	0.823	52 (48.1)	56 (51.9)	0.675	0.425	43 (39.8)	65 (60.2)		
Married	90 (49.7)	91 (50.3)			142 (50.5)	139 (49.5)			142 (50.5)	139 (49.5)		
**Housing ownership**												
Owner	73 (66.4)	37 (33.6)	0.952	0.725	79 (58.5)	56 (41.5)	**0.015**	**0.010**	76 (56.3)	59 (43.7)	**0.041**	**0.016**
Others	142 (66.7)	71 (33.3)			110 (44.9)	135 (55.1)			112 (45.7)	133 (54.3)		

HC, Hip circumference; FBS, Fasting blood sugar; TC, Total cholesterol; TG, Triglyceride; HDL, High-density lipoprotein; LDL, Low-density lipoprotein; hs-CRP, High-sensitivity C-reactive protein; Il-1β, Interleukin-1β; TAC, Total antioxidant capacity; BMI, Body mass index; BFM, Body fat mass; FFM, Fat-free mass; FFMI, Fat-free mass index; FMI, Fat mass index; WC, Waist circumference; NC, neck circumference; WHR, Waist hip ratio; VFL, Visceral fat level.

Values are mean ± SD for crude model and mean ± SE for adjusted model and qualitative variables are presented as n (%).

All variables adjusted with age, energy intake, physical activity, BMI. BMI consider as collinear variable for body composition and anthropometric measurements.

The crude p-value was obtained from one-way analysis of variance (ANOVA).

† Adjust p-value obtained from an analysis of covariance (ANCOVA).

*P* < 0.05 consider as significant, P = 0.05–0.07 consider as marginally significant.

Bold values means significant p-value *P* < 0.05.

#### General characteristics of participants among processed meat intake categories

Among processed meat categories, there was a significant mean difference for SBP (*p* < 0.001). But, significance was lost after adjusting for potential confounders (*p* = 0.130). In the adjusted model, in body composition variables, there were significant mean differences for SMM (*p* = 0.054), FFM (*p* = 0.030), SLM (*p* = 0.022), and trunk fat (*p* = 0.041), FMI (*p* = 0.062) and in categorical variables; economic status (*p* = 0.011) (Table 1).

#### General characteristics of participants among red meat intake categories

There was a significant difference in economic status (*p* = 0.0003), education level (*p* = 0.002), and housing ownership (*p* = 0.010) before and after adjustment Table 1. Moreover, the crude model showed a marginally significant difference between groups for BFM (*p* = 0.071). High adherence to red meat was associated with lower levels of TG (*p* = 0.023) and WC (*p* = 0.034). No significant differences were noted in other variables (*p* > 0.05) (Table 1).

#### General characteristics of participants among white meat intake categories

After adjusting for potential confounders, there was a significant marginal difference in BMI (*p* = 0.072). There were also significant differences in economic status (*p* = 0.002), education level (*p* = 0.001), and housing ownership (*p* = 0.016). Women with a higher intake of white meat had marginally significantly higher mean HDL (*p* = 0.070), total cholesterol (*p* = 0.030), and lowered FBS (*p* = 0.041), but these associations were not present after controlling confounders (Table 1).

#### Dietary intake of the study population across intakes of red, white, and processed meat

The dietary intake of the participants across two groups to intakes of red meat, white meat, and processed meat is shown in Table 2. The mean energy intake in low and high-intake processed meat was 2,763.591 vs. 2,447.06 kcal/day, respectively, and was statistically significant (*p* = 0.002). Additionally, the intake of energy was high in low intake white meat, 2,695 vs. 2,495.27 gr with (*p* = 0.053). After adjustment for confounders (including age, BMI, physical activity, and total energy intake) mean of red meat, protein, vegetables, saturated fatty acid (SFA), eicosapentaenoic acid (EPA), Docosahexaenoic acid (DHA), zinc, copper, potassium, vitamins of C, A, B3, B6, and B12 consumption was higher in the upper median group of red meat (*p* ≤ 0.05). In contrast, intake of total fiber, linoleic acid, polyunsaturated fatty acids (PUFA), and vitamin E were low in participants with high red meat intake (*p* ≤ 0.05). Our results also showed that sodium and vitamin B12 were significantly higher (*p* ≤ 0.05) in subjects with increased consumption of processed meat compared to those with low intake. Protein, vegetables, EPA, DHA, zinc, calcium, potassium, and vitamins of A, B3, B6, B2, and B12 consumption was greater in those with high white meat intake (*p* < 0.05). On the other hand, fat, monounsaturated fatty acids (MUFA), linoleic acid, PUFA, Manganese, and vitamin E consumption was low in participants with high white meat intake (*p* ≤ 0.05).

**Table 2:** Dietary intakes of study population between medians of processed meat, red meat, and white meat in 391 obese and overweight women.

**Variables**	**Processed meat median**		***p*** **-** **value^†^**	**Red meat median**		***p*** **-** **value^†^**	**White meat median**		***p*** **-** **value^†^**
	**Low (<19.82)**	**High (>19.83)**		**Low** **(<36.46)**	**High** **(>36.47)**		**Low** **(<31.02)**	**High** **(>31.03)**	
	**Mean** ±**SD**			**Mean** ±**SD**			**Mean** ±**SD**		
**Food groups**									
Cereal (g/d)	448.280 ± 18.741	417.501 ± 18.744	0.330	433.401 ± 17.332	427.391 ± 18.180	0.261	435.962 ± 18.321	425.681 ± 17.310	0.272
Whole grain (g/d)	106.130 ± 5.842	91.011 ± 5.845	0.370	105.485 ± 5.264	92.812 ± 5.520	0.120	95.020 ± 5.587	483.766 ± 18.403	0.164
Refined grain (g/d)	490.500 ± 19.628	474.842 ± 19.623	0.544	481.190 ± 16.422	468.492 ± 17.435	0.602	491.751 ± 18.401	473.090 ± 17.383	0.127
Fruits (g/d)	680.850 ± 30.234	622.445 ± 30.231	0.332	659.170 ± 26.221	677.145 ± 27.504	0.376	641.784 ± 27.700	681.055 ± 26.164	0.162
Vegetables (g/d)	470.861 ± 22.652	378.493 ± 22.654	0.167	408.146 ± 20.990	473.478 ± 22.022	**0.005**	389.584 ± 21.977	483.762 ± 20.762	**0.001**
Legumes (g/d)	106.035 ± 4.361	99.832 ± 4.361	0.29	102.941 ± 3.693	100.970 ± 3.874	0.722	102.476 ± 3.900	101.585 ± 3.694	0.570
Nuts (g/d)	37.271 ± 1.240	34.864 ± 1.874	0.284	13.220 ± 1.432	15.651 ± 1.522	0.268	14.700 ± 1.505	14.103 ± 1.465	0.775
Dairy (g/d)	346.560 ± 22.191	333.133 ± 22.191	**0.04**	327.064 ± 22.330	346.383 ± 23.001	0.550	313.610 ± 24.647	360.154 ± 23.684	0.173
Eggs (g/d)	26.501 ± 1.171	25.634 ± 1.760	0.680	21.011 ± 1.352	21.306 ± 1.431	0.882	26.099 ± 1.384	26.190 ± 1.341	0.885
White Meat (g/d)	64.555 ± 4.360	74.456 ± 6.570	0.702	40.564 ± 4.789	54.003 ± 5.075	0.080	35.402 ± 4.113	87.905 ± 3.987	**<0.001**
Red meat (g/d)	23.290 ± 1.450	21.032 ± 2.182	0.397	9.261 ± 1.070	33.55 ± 1.144	**<0.001**	36.790 ± 1.695	41.933 ± 1.642	**0.050**
Processed meat (g/d)	4.351 ± 0.974	26.635 ± 1.466	**0.001**	10.987 ± 1.536	11.433 ± 1.631	0.845	17.237 ± 1.570	18.881 ± 1.523	0.430
**Energy and macronutrients**									
Energy (kcal/d)	2,763.590 ± 71.711	2,447.060 ± 71.714	**0.002**	2,644.850 ± 66.642	2,522.055 ± 68.653	0.201	2,695.801 ± 74.656	2,495.270 ± 71.751	**0.053**
Carbohydrates (g/d)	370.852 ± 42.570	330.475 ± 46.580	0.092	375.291 ± 4.268	367.676 ± 4.475	0.560	369.344 ± 4.985	366.442 ± 4.838	0.634
Fat (g/d)	99.930 ± 0.515	112.695 ± 0.395	0.264	94.150 ± 1.822	93.864 ± 1.910	0.535	98.29 ± 2.1443	91.471 ± 2.084	**0.030**
protein (g/d)	92.001 ± 0.212	93.284 ± 0.166	0.327	86.300 ± 1.543	92.826 ± 1.614	**0.002**	81.420 ± 1.665	97.835 ± 1.610	**<0.001**
Total fiber (g/d)	45.930 ± 1.184	42.451 ± 1.776	0.101	47.49 ± 1.432	42.18 ± 1.430	**0.008**	44.68 ± 1.435	45.02 ± 1.398	0.917
MUFA (g/d)	31.252 ± 0.814	33.020 ± 1.211	0.222	32.565 ± 0.933	30.921 ± 0.995	0.233	33.360 ± 0.942	30.372 ± 0.919	**0.032**
PUFA (g/d)	20.06 ± 0.690	21.200 ± 1.034	0.364	22.200 ± 0.774	18.400 ± 0.821	**0.001**	22.173 ± 0.791	18.715 ± 0.790	**0.004**
SFA (g/d)	27.615 ± 0.685	28.789 ± 1.037	0.340	26.961 ± 0.782	29.296 ± 0.837	**0.044**	28.195 ± 0.844	28.000 ± 0.000	0.981
TFA (g/d)	0.001 ± 0.000	0.001 ± 0.000	0.262	0.001 ± 0.000	0.001 ± 0.000	0.531	98.07 ± 2.194	91.350 ± 2.134	**0.064**
Linolenic acid (g/d)	1.22 ± 0.041	1.184 ± 0.076	0.684	1.197 ± 0.052	1.231 ± 0.064	0.650	1.243 ± 0.055	1.187 ± 0.054	**<0.001**
Linoleic acid (g/d)	17.313 ± 0.681	18.440 ± 1.032	0.366	19.633 ± 0.762	15.440 ± 0.811	**<0.001**	19.931 ± 0.794	15.510 ± 0.773	0.420
EPA (g/d)	0.035 ± 0.000	0.034 ± 0.000	0.860	0.022 ± 0.000	0.041 ± 0.000	**0.016**	0.010 ± 0.000	0.051 ± 0.000	**<0.001**
DHA (g/d)	0.110 ± 0.010	0.129 ± 0.015	0.585	0.093 ± 0.010	0.13 ± 0.011	**0.015**	0.053 ± 0.012	0.170 ± 0.015	**<0.001**
**Micronutrients**									
Iron (mg/d)	18.880 ± 1.521	18.14 ± 0.36	0.090	18.544 ± 0.271	18.776 ± 0.293	0.581	18.484 ± 0.291	18.844 ± 0.282	0.451
Zinc (mg/d)	12.910 ± 0.184	12.866 ± 0.274	0.891	12.286 ± 0.202	13.584 ± 0.217	**<0.001**	12.154 ± 0.2152	13.280 ± 0.210	**0.011**
Copper (mg/d)	2.00 ± 0.035	1.94 ± 0.044	0.295	1.940 ± 0.031	2.041 ± 0.034	**0.046**	1.950 ± 0.034	2.021 ± 0.035	0.204
Calcium (mg/d)	1,162.161 ± 32.742	1,172.45 ± 26.406	0.834	1,166.026 ± 30.483	1,164.521 ± 32.34	0.970	1,111.252 ± 30.720	1,224.595 ± 29.775	**0.015**
Magnesium (mg/d)	462.410 ± 6.674	446.394 ± 10.055	0.185	450.163 ± 7.712	463.011 ± 8.191	0.265	448.360 ± 8.021	465.145 ± 7.775	0.165
Potassium (mEq/d)	4,264.320 ± 84.152	4,197.241 ± 126.681	0.664	4,079.702 ± 95.605	4,428.041 ± 101.455	**0.014**	4,066.015 ± 99.734	4,412.294 ± 97.071	**0.016**
Manganese (mg/d)	7.160 ± 0.155	6.634 ± 0.231	**0.061**	7.164 ± 0.185	6.824 ± 0.195	0.205	7.335 ± 0.185	6.701 ± 0.186	**0.015**
Sodium (mg/d)	4,120.562 ± 93.875	4,560.734 ± 141.312	**0.015**	4,350.365 ± 109.855	4,149.211 ± 116.558	0.214	4,226.274 ± 115.031	4,283.491 ± 111.975	0.721
Vitamin C (mg/d)	185.302 ± 7.992	180.140 ± 12.031	0.721	169.271 ± 9.092	199.950 ± 9.655	**0.020**	174.865 ± 9.705	192.964 ± 9.391	0.095
Vitamin E (mg/d)	17.732 ± 0.811	18.850 ± 1.230	0.456	20.351 ± 0.912	15.521 ± 0.974	**<0.001**	20.404 ± 0.931	15.815 ± 0.904	**0.001**
Vitamin A (mg/d)	781.430 ± 29.713	760.85 ± 44.732	0.702	707.300 ± 33.542	851.30 ± 35.60	**0.004**	698.516 ± 34.612	855.571 ± 33.544	**0.001**
Thiamin (mg/d)	2.10 ± 0.034	2.07 ± 0.045	0.634	2.134 ± 0.034	2.054 ± 0.032	0.120	2.081 ± 0.035	2.114 ± 0.031	0.654
Riboflavin (mg/d)	2.16 ± 0.043	2.19 ± 0.060	0.616	2.155 ± 0.054	2.197 ± 0.056	0.551	2.060 ± 0.052	2.275 ± 0.042	**0.003**
Niacin (mg/d)	25.25 ± 0.547	26.18 ± 0.810	0.345	24.585 ± 0.616	26.604 ± 0.651	**0.025**	22.480 ± 0.560	28.330 ± 0.541	**<0.001**
Vitamin B6 (mg/d)	2.16 ± 0.031	2.13 ± 0.021	0.654	2.041 ± 0.041	2.292 ± 0.044	**<0.001**	1.971 ± 0.042	2.341 ± 0.042	**<0.001**
Folate (mcg/d)	606.990 ± 9.032	582.53 ± 13.591	0.131	643.004 ± 8.985	603.032 ± 10.484	0.621	599.32 ± 10.881	603.501 ± 10.540	0.763
Vitamin B12 (mcg/d)	4.193 ± 0.185	4.95 ± 0.276	**0.020**	3.771 ± 0.201	5.161 ± 0.211	**<0.001**	3.87 ± 0.200	4.962 ± 0.205	**<0.001**

MUFA, monounsaturated fatty acid; PUFA, polyunsaturated fatty acid; SFA, saturated fatty acid; TFA, trans fatty acid; EPA, Eicosapentaenoic acid; DHA., Docosahexaenoic acid.

Values are mean ± SD obtained from one-way analysis of variance (ANOVA).

Nutrients and food groups were adjusted for age, BMI, physical activity and total energy intake. P-value for energy intake obtained from controlling age,BMI, and physical activity.

† Adjust p-value obtained from an analysis of covariance.

*P* < 0.05 consider as significant, P = 0.05–0.07 consider as marginally significant.

Bold values means significant p-value *P* < 0.05.

### Inflammatory biomarkers among medians of red, white, and processed meat

There was no significant difference in PAI-1, Gal-3, hs-CRP, or MCP-1 between low and high processed meat intake categories before adjustment (*p* > 0.05), but in model 2 that adjusted for potential confounder, mean PAI-1 (*p* = 0.052), Gal-3 (*p* = 0.054), hs-CRP (*p* = 0.050), MCP-1 (*p* = 0.046) was higher in a participant with high adherence of proceeded meat, Table 3. The mean of HOMA-IR (*p* = 0.045) and IL1b (*p* = 0.071) was low in women with a high intake of red meat in models 1 and 2. However, in the crude model mean of HOMA-IR was high in subjects with a high white meat intake (*p* = 0.024), but in model 2 mean of its index was low in subjects with a high intake of white meat with no significance (*p* = 0.610). The mean of leptin was higher in those with a low intake of white meat in the crude model (*p* = 0.882); after adjustment, this association remained significant (*p* = 0.024), (Table 3).

**Table 3:** Inflammatory biomarkers between low and high category of processed meat, red meat and white meat (g/d) in 391 obese and overweight women.

**Variables**		**Processed meat median**		***P*** **-value**	***P*** **-value^†^**	**Red meat median**		***P*** **-value**	***P*** **-value**	**White meat median**		***P*** **-value**	***P*** **-value^†^**
		**Low (<19.82)**	**High** **(>19.83)**			**Low (<36.46)**	**High** **(>36.47)**			**low (<31.02)**	**High** **(>31.03)**		
HOMA-IR	Crude	3.321 ± 1.411	3.172 ± 1.215	0.435		3.480 ± 1.400	3.201 ± 1.122	0.091		3.144 ± 1.215	3.530 ± 1.313	**0.024**	
	Model 1	3.000 ± 00.315	2.921 ± 0.412		0.884	3.489 ± 0.261	2.626 ± 0.291		**0.045**	3.303 ± 0.284	2.865 ± 0.291		0.301
	Model 2	2.937 ± 0.334	3.026 ± 0.443		0.882	3.511 ± 0.340	2.274 ± 0.392		**0.045**	3.104 ± 0.341	2.927 ± 0.374		0.610
Leptin (mg/L)	Crude	26.301 ± 12.192	29.932 ± 11.170	0.164		27.436 ± 11.970	27.952 ± 11.922	0.846		27.894 ± 11.553	12.310 ± 1.832	0.882	
	Model 1	25.923 ± 1.451	29.670 ± 1.865		0.124	27.184 ± 1.653	27.546 ± 1.616		0.884	27.640 ± 1.651	27.129 ± 1.581		0.821
	Model 2	26.532 ± 1.475	28.711 ± 1.920		0.391	27.763 ± 1.642	27.004 ± 1.606		0.754	29.156 ± 1.654	25.660 ± 1.572		**0.024**
PAI1 (mg/L)	Crude	17.081 ± 30.690	17.481 ± 30.695	0.950		12.845 ± 23.124	18.876 ± 34.841	0.201		13.580 ± 22.321	18.755 ± 36.312	0.270	
	Model 1	7.920 ± 9.134	37.592 ± 12.041		**0.07**	15.574 ± 8.116	17.651 ± 8.970		0.860	13.722 ± 8.294	19.587 ± 8.723		0.631
	Model 2	6.282 ± 9.231	40.230 ± 12.313		**0.052**	23.16 ± 11.055	14.33 ± 12.840		0.641	12.665 ± 10.310	26.690 ± 11.012		0.380
Gal-3 (mg/L)	Crude	4.946 ± 1.320	5.594 ± 2.035	0.782		23.160 ± 11.051	14.332 ± 12.842	0.660		3.865 ± 5.867	4.665 ± 9.161	0.642	
	Model 1	2.065 ± 1.532	6.961 ± 2.021		0.080	3.040 ± 1.396	3.874 ± 1.542		0.690	3.191 ± 1.421	3.66 ± 1.500		0.820
	Model 2	1.800 ± 1.532	7.381 ± 2.040		**0.054**	4.371 ± 1.832	3.396 ± 2.132		0.750	3.100 ± 1.721	4.885 ± 1.833		0.511
Hs-CRP (mg/ L)	Crude	4.622 ± 5.055	3.675 ± 3.771	0.220		4.540 ± 4.566	3.901 ± 4.575	0.284		4.211 ± 4.850	4.215 ± 4.270	0.991	
	Model 1	2.531 ± 0.600	4.341 ± 0.791		0.091	5.574 ± 0.724	3.060 ± 0.801		**0.031**	4.435 ± 0.795	4.441 ± 0.834		0.990
	Model 2	2.550 ± 0.622	5.31 ± 0.831		**0.050**	5.046 ± 0.775	3.384 ± 0.902		0.220	4.152 ± 0.754	4.494 ± 0.811		0.773
IL1b (mg/L)	Crude	0.400 ± 0.532	0.301 ± 0.565	0.280		0.422 ± 0.492	0.321 ± 0.562	0.130		0.370 ± 0.535	0.375 ± 0.522	0.941	
	Model 1	0.476 ± 0.091	0.176 ± 0.425		0.081	0.540 ± 0.080	0.172 ± 0.099		**0.005**	0.410 ± 0.094	0.333 ± 0.091		0.580
	Model 2	0.462 ± 0.91	0.18 ± 0.135		0.130	0.517 ± 0.107	0.16 ± 0.1271		**0.071**	0.394 ± 0.105	0.315 ± 0.119		0.645
MCP-1 (mg/L)	Crude	56.494 ± 96.055	56.340 ± 111.661	0.991		51.200 ± 104.101	50.901 ± 81.830	0.98		52.281 ± 95.400	49.815 ± 91.681	0.842	
	Model 1	32.654 ± 22.971	108.901 ± 30.275		**0.071**	44.100 ± 21.360	63.850 ± 23.625		0.551	54.591 ± 22.003	51.324 ± 23.131		0.921
	Model 2	29.584 ± 22.921	113.874 ± 30.565		**0.046**	64.051 ± 27.554	58.971 ± 32.001		0.910	54.743 ± 25.940	69.732 ± 27.704		0.711

Gal-3, Galectin 3; Hs-CRP, high-sensitivity C-reactive protein; HOMA-IR, homeostasis model assessment of insulin resistance; IL-1b, interleukin 1 beta; MCP-1, chemoattractant protein-1; PA-1, plasminogen activator inhibitor-1; TGF-β, transforming growth factor.

Values are mean ± SD for the crude model and mean ± SE for an adjusted model.

Model 1: Adjusted for: age, BMI, physical activity, total energy intake, economic status, education, housing ownership.

Model 2: Additionally controlled for vegetables and dairy for processed meat.

The crude p-value for all variables obtained from the one-way analysis of variance (ANOVA).

† Adjust p-value obtained from an analysis of covariance.

*P* < 0.05 consider as significant, P = 0.05–0.07 consider as marginally significant.

Bold values means significant p-value *P* < 0.05.

### Association between red, white as well as processed meat and inflammatory biomarkers

In the crude model, there was a significant association between HOMA-IR and processed meat (β: 0.410, 95% CI: 0.070;0.750, *p* = 0.011), but after adjusting for potential confounders, in model 3 this association disappears (β: −0.007, 95% CI: −0.021;0.007, *p* = 0.384). Result in the crude model showed that increased intake of processed meat had a significant positive association with the level of leptin (β: 0.172, 95% CI:0.020;0.321, *p* = 0.021) and Gal-3 (β: 0.052, 95% CI: 0.001,0.205, *p* = 0.031) that after adjustment in model 3 it remained significant (β: 0.900, 95% CI: 0.031;1.233, *p* = 0.015). A positive association with the level of MCP-1 (β:17.287, 95% CI: −12.692;47.267, *p* = 0.250) was not statistically significant, but after considering the confounding factors, a significant relationship was observed (β:0.304, 95% CI:0.100;1.596, *p* = 0.025) Table 4. Moreover, a positive significant association between the level of hs-CRP, PAI-1 and MCP1 and high adherence to red meat (β:0.020, 95% CI:0.001;0.050, *p* = 0.014), (β:0.263, 95% CI:0.112;0.345, *p* = 0.053), (β:0.490, 95% CI: 0.175;1.464, *p* = 0.071) and inverse association with HOMA-IR (β: −0.016, 95% CI: −0.022, −0.001, *p* = 0.033) in model 3 was established. Our analysis demonstrated a significantly negative association between levels of Gal-3 and MCP-1 and high adherence to white meat (β: −0.110, 95% CI: −0.271;0.000, *p* = 0.044) and (β: −1.933, 95% CI: −3.721;0.192, *p* = 0.022) after adjustment for confounder. In contrast, a significant positive association between the level of PAI-1 and high adherence to white meat (β: 0.154, 95% CI: 0.021;0.283, *p* = 0.028) was observed in the crude model, but after adjustment, this association reached marginal significance (β: −0.340, 95% CI: −0.751;0.050, *p* = 0.070). After controlling potential confounders, there was a marginally significant inverse association between higher intake of white meat with HOMA-IR (β: −0.011, 95% CI: −0.020,0.000, *p* = 0.070) (Table 4).

**Table 4:** Association between inflammatory biomarkers with processed meat, red meat, and white meat (g/d) in 391 obese and overweight women.

**Variables**		**Processed meat**			***p*** **-value**	***p*** -value^*^	**Red meat**			***p*** **-value**	***p*** -value^*^	**White meat**			***p*** **-value**	***p*** -value^*^
		**β**	**CI (95%)**	**R^2^**			**β**	**CI (95%)**	**R^2^**			**β**	**CI (95%)**	**R^2^**		
HOMA-IR	**Crude**	0.410	0.070, 0.750	0.003	**0.011**		−0.008	−0.011, 0.001	0.013	**0.070**		0.002	−0.002, 0.007	0.012	0.320	
	**Model 1**	−0.003	−0.01, 0.01	0.110		0.674	−0.008	−0.011, 0.000	0.526		**0.071**	0.000	−0.004, 0.005	0.011		0.705
	**Model 2**	−0.007	−0.022,0.007	0.536		0.322	−0.008	−0.014,0.0021	0.011		**0.061**	0.001	−0.003,0.005	0.120		0.601
	**Model 3**	−0.007	−0.021, 0.000	0.570		0.381	−0.016	−0.022, −0.001	0.120		**0.033**	−0.011	−0.020, 0.000	0.240		**0.070**
Leptin (mg/L)	**Crude**	0.172	0.020, 0.321	0.240	**0.021**		−0.070	−0.171, 0.030	0.133	0.172		−0.030	−0.100, 0.040	0.450	0.445	
	**Model 1**	0.145	−0.030, 0.235	0.457		0.151	−0.060	−0.15, 0.030	0.526		0.195	−0.021	−0.090, 0.041	0.520		0.485
	**Model 2**	0.780	0.031, 0.855	0.655		**0.040**	0.000	−0.114, 0.030	0.625		0.222	−0.010	−0.09, −0.00	0.670		**0.066**
	**Model 3**	0.900	0.031, 1.233	0.750		**0.015**	0.000	−0.114, 0.030	0.903		0.403	−0.010	−0.09, −0.00	0.020		**0.080**
PAI1 (mg/L)	**Crude**	3.765	−8.132, 15.650	0.010	0.535		0.274	0.014, 0.540	0.040	**0.040**		0.154	0.021, 0.283	0.107	**0.028**	
	**Model 1**	−0.020	−0.450,0.40	0.010		0.901	0.311	0.026,0.693	0121		**0.030**	0.102	−0.440,1.054	0.100		0.126
	**Model 2**	−0.071	−0.521, 0.384	0.110		0.755	0.240	−0.030, 0.555	0.124		0.080	0.100	−0.030, 0.301	0.150		0.145
	**Model 3**	−0.140	−0.580, 0.296	0.140		0.511	0.263	0.112, 0.345	0.144		**0.053**	−0.340	−0.751, 0.050	0.161		**0.070**
Gal-3 (mg/L)	**Crude**	2.025	−2.33, 6.370	0.010	0.352		0.012	−0.115, 0.152	0.010	0.770		0.010	−0.055, 0.071	0.020	0.720	
	**Model 1**	0.302	−0.121, 0.180	0.070		0.690	−0.011	−0.141, 0.110	0.140		0.781	−0.020	−0.09, 0.04	0.110		0.500
	**Model 2**	0.011	−0.145, 0.184	0.112		0.830	−0.009	−0.145, 0.121	0.141		0.892	−0.03	−0.10, 0.03	0.110		0.360
	**Model 3**	0.052	0.001, 0.205	0.178		**0.031**	−0.030	−0.210, 0.200	0.172		0.788	−0.110	−0.271, −0.000	0.170		**0.044**
Hs-CRP (mg/L**)**	**Crude**	−0.011	−0.072,0.031	0.004	0.485		0.015	−0.020,0.041	0.004	0.455		0.005	−0.015,0.021	0.000	0.490	
	**Model 1**	−0.020	−0.071, 0.025	0.160		0.300	0.021	−0.005, 0.06	0.150		0.092	0.001	−0.010, 0.015	0.157		0.360
	**Model 2**	0.010	−0.071, 0.020	0.190		0.321	0.030	0.000, 0.060	0.190		**0.040**	0.001	−0.010, 0.060	0.186		0.264
	**Model 3**	0.010	−0.065, 0.040	0.198		0.732	0.020	0.001, 0.050	0.193		**0.014**	0.056	0.000, 0.110	0.188		0.445
IL1b (mg/L)	**Crude**	−0.003	−0.147, 0.146	0.050	0.990		0.001	−0.003, 0.005	0.04	0.680		0.000	−0.001, 0.002	0.04	0.590	
	**Model 1**	−0.003	−0.009, 0.003	0.112		0.320	0.001	−0.004, 0.005	0.135		0.510	0.000	−0.002, 0.002	0.110		0.917
	**Model 2**	−0.002	−0.009, 0.004	0.175		0.442	0.002	−0.002, 0.006	0.140		0.400	0.000	−0.002, 0.002	0.155		0.854
	**Model 3**	−0.004	−0.010, 0.000	0.190		0.175	0.000	−0.007, 0.006	0.250		0.931	0.002	−0.005, 0.009	0.189		0.615
MCP-1 (mg/L)	**Crude**	17.287	−12.692, 47.267	0.040	0.250		0.251	−0.494, 1.001	0.040	0.501		0.135	−0.20, 0.47	0.01	0.435	
	**Model 1**	−0.142	−1.321, 1.024	0.010		0.800	0.382	−0.481, 1.250	0.120		0.381	0.081	−0.265, 0.432	0.114		0.611
	**Model 2**	−0.220	−1.442, 1.001	0.120		0.721	0.365	−0.500, 1.221	0.135		0.410	0.045	−0.861, 1.200	0.140		0.743
	**Model 3**	0.304	0.100, 1.596	0.139		**0.025**	0.490	0.175, 1.464	0.140		**0.071**	−1.933	−3.721, −0.192	0.141		**0.022**

Gal-3, Galectin 3; Hs-CRP, high-sensitivity C-reactive protein; HOMA-IR, homeostasis model assessment of insulin resistance; IL-1b, interleukin 1 beta; MCP-1, chemoattractant protein-1; PA-1, plasminogen activator inhibitor-1; R^2^, r squared; TGF-β, transforming growth factor.

Values are: beta (β), 95% confidence interval (CI).

Model 1: Adjusted for: age, BMI, physical activity, total energy intake.

Model 2: Additionally controlled for economic status, education, housing ownership.

Model 3: Additionally controlled for vegetables intake and dairy for processed meat for red meat and white meat group.

* *P*-value obtained from adjustment. All p-values were obtained from linear regression.

*P* < 0.05 consider as significant, P = 0.05–0.07 consider as marginally significant.

Bold values means significant p-value *P* < 0.05.

## Discussion

In this study, we evaluated the relationship between the consumption of red, white, and processed meats with inflammatory and metabolic biomarkers in overweight and obese women. We found that red meat intake was positively associated with PAL-1, hs-CRP, and MCP-1 levels. Moreover, processed meat intake was positively associated with different biomarkers such as leptin, Gal-3, and MCP-1. On the contrary, negative relationships between high adherence to white meat intake and Gal-3, MCP-1, PA1, and HOMA-IR were established.

In the current cross-sectional study, greater processed meat intakes were initially positively associated with higher HOMA-IR and leptin levels. However, HOMA-IR was no longer associated with processed meat intake after adjustment for all confounders. After controlling for all confounders in the last model of adjustment, there was a positive association between processed meat intake and levels of leptin, Gal-3, and MCP-1. In line with our study, a cohort study displayed that processed meat consumption was positively associated with leptin and CRP in both men and women after 9 years of follow-up (41). Another study expressed that higher processed meat consumption is positively associated with inflammatory markers in 403,886 British adults (42). That seems the binding capacity of iron in our body might be exceeded by consuming processed meat containing high amounts of heme iron. Oxidative stress can be increased by free iron and, as a result, act as a proinflammatory agent (43).

In our study, consumption of white meat was negatively associated with HOMA-IR, Gal-3, MCP-1, and PAL-1. In line with these findings, several studies have reported that higher consumption of white meat such as poultry has been related to lower inflammatory markers such as CRP (44–46). The consumption of fish and seafood, considered white meat, was shown to have anti-inflammatory properties due to the high contents of omega-3 fatty acids and lesser amount of heme iron and lower cholesterol (47–49).

Our results also showed a significant positive association between red meat intake and levels of PAL-1, hs-CRP, and MCP-1; however, we observed a negative association between red meat consumption and HOMA-IR. Similar to our results, in two separate studies, including 482 Iranian women and 2,500 German individuals (both studies included healthy adults), greater red meat intake was associated with a higher plasma CRP concentration (50, 51). However, in a Dutch study, only processed meat intake was associated with higher CRP, while red meat intake was not (52). In a clinical trial, replacing energy from carbohydrates with protein from unprocessed lean red meat for 8 weeks did not augment inflammatory marker concentrations (19). In a randomized cross-over controlled trial in 36 individuals aged 39–97 years (53), 5 weeks of dietary treatment with 28, 113, or 153 g of lean red meat per day had similar advantageous effects on LDL-c and similar disadvantages effects on HDL-c. The authors include lean red meat in a diet to reduce cardiovascular disease (CVD) risk (53). This discrepancy with our study can be attributed to methodological or ethnic differences. In Iran, per capita, meat consumption is around 35.5 kg/year, comprising 30 gr of red meat and 60 gr of white meat per day (54). In our study, the average consumption of red meat is generally low compared to other countries such as 136 gr/d in 2021 in the united states (55). Indeed, that seems obese/overweight women in this study population even with a higher than the median red meat intake have just reached the ordinary intake of red meat intake like other populations (54, 55). Additionally, participants with higher red meat intake in the current study also had higher BFM levels and higher vegetable intakes; hence, all this information should be considered when interpreting our results. However, it seems that the mechanism is not completely clear and there is a need to design studies for further investigations. Monton et al. evaluated the association between the consumption of red meat and circulating hs-CRP levels in 2,198 men and women and expressed a positive association in these values (50). A diet high in red and processed meat can contribute to weight gain and body fat accumulation, which induces the obesity-related inflammatory process (41). However, decreasing body fat might be more relevant than lowering the intake of dietary red and processed meat to improve circulating levels of adipokines and inflammatory markers (31).

Iron may be another component of red meat contributing to the progression of metabolic abnormalities (56–58). Indeed, iron is a prooxidant that has been related to increased oxidative stress (59). High iron contents of red meat, especially heme iron, and meat processing particularly through high-temperature cooking, results in the formation of carcinogenic chemicals, including N-nitroso-compounds (NOC) and polycyclic aromatic hydrocarbons (PAH) (60, 61). Since inflammation is a key risk factor for metabolic diseases, our results may suggest that high red and processed meat consumption can affect metabolic disease development through the inflammatory pathway. Dietary intake of heme iron, abundant in red meat, has also been related to increased CVD risk especially inflammation (62), likely through inflammation and lipid peroxidation mechanisms.

A recent study reported that increased red meat consumption is associated with higher mortality risk in both women and men (63). Mercapturic acid of dihydroxynonane (DHN-MA), a substance that indicates the production of hydroxynonenal (HNE) in food or during digestion, is excreted in large amounts by diets that include heme iron and −6 fatty acids, such as hemoglobin or red meat and safflower oil (64, 65). Foods containing heme iron and PUFAs also contain HNE (66). The HNE, an end product of the oxidative breakdown of −6 fatty acids and a well-known bioactive marker of lipid peroxidation involved in cell growth control and signaling, is the main urinary metabolite of HNE, and it is also known as DHN-MA. As a result, DHN-MA has also been employed as an oxidative stress marker (67, 68).

**Figure 1:**
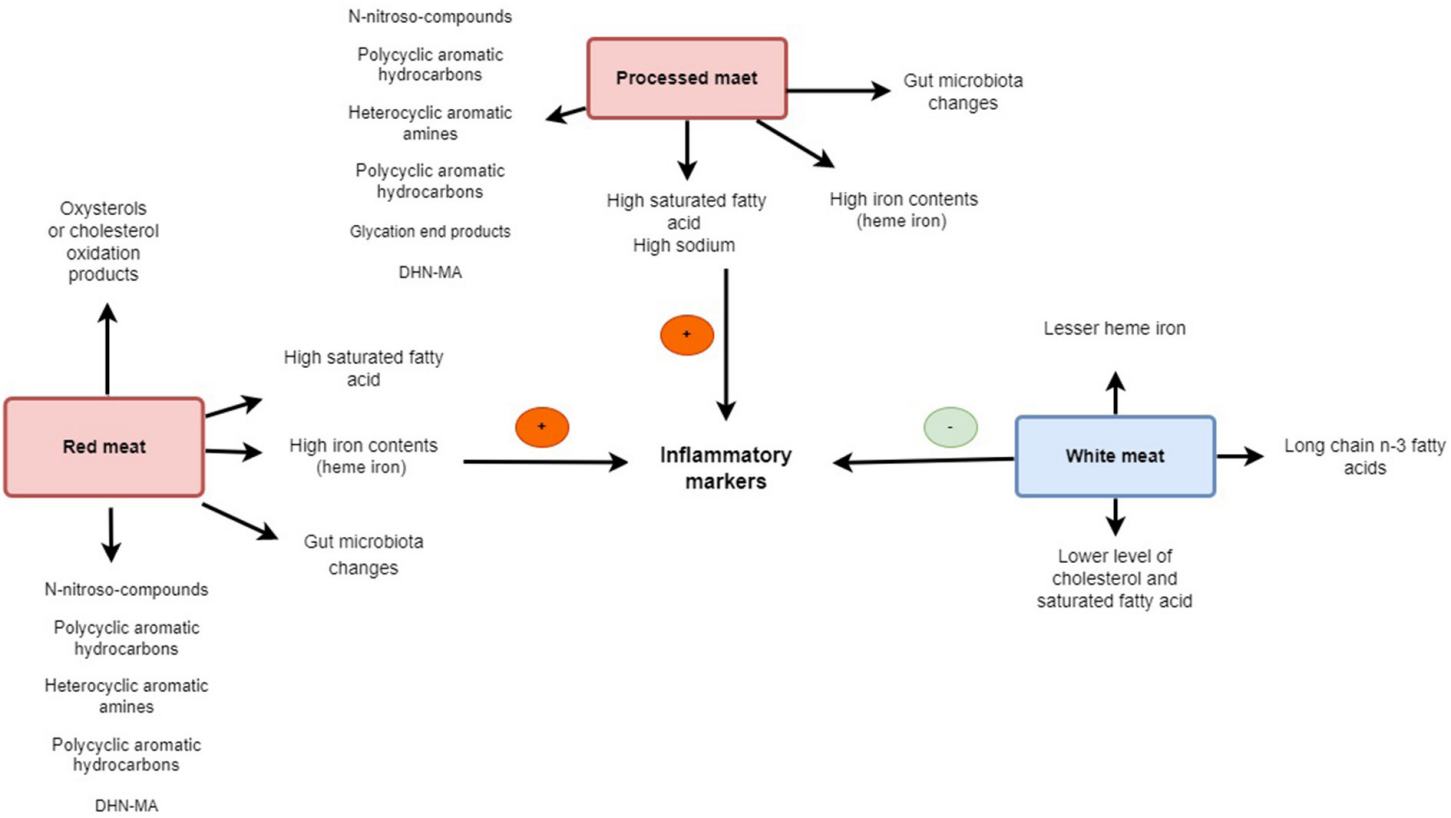
The scheme illustrates the different mechanisms of red, processed, and white meat on inflammation.

It has been expressed that the red meat dietary intervention resulted in significant increments in plasma concentrations of trimethylamine-N-oxide (TMAO) (69) which has been linked to inflammation and CVD (70, 71). TMAO may cause inflammation. The inflammatory cytokines IL-1 and IL-18 were generated by the TXNIP-NLRP3 inflammasome after TMAO dramatically increased oxidative stress and activated it, but it also reduced the generation of nitric oxide (NO) and endothelial nitric oxide synthase (eNOS) (72). In addition preservatives such as sodium, nitrates, advanced glycation end products (AGEs), and their by-products may contribute to the relationship between red meat intake and increment of inflammation (73–75). Exposure to high temperatures can generate high levels of AGEs in meat, which have been shown to augment oxidative and inflammatory processes (73). Christ et al. (76) identified a functional role for NLRP3/IL-1β in the induction of innate immune memory in monocytes as triggered by a high intake of red meat and represented how this promotes inflammatory diseases (76). Apart from the meat cooking method of meat, enhancing flavor or improving preservation through methods such as smoking, salting, curing, or even adding chemical preservatives may affect oxidative stress and inflammation status (60, 77). The iron content in red meat has been related to upregulating inflammatory mediators, such as IL-6, IL-1β, and tumor necrosis factor-alpha (TNF-α) (78). Moreover, another proposed mechanism in the relationship between meat consumption and inflammation is probably related to the gut microbiome. It has also been displayed that red meat's gut microbiota-dependent breakdown metabolic processes can trigger inflammatory disease (70, 79). The most mechanistic relation of all kinds of meat on inflammation is present in Figure 1.

The present study has several strengths and limitations. First, this is the first study that evaluated the association between red, white, and processed meat on inflammatory and metabolic biomarkers in overweight and obese women. Second, dietary intake was assessed using a validated FFQ, which an experienced dietitian completed to decrease measurement errors. Anthropometric indices and body composition outcomes were assessed by the same person each time to improve the accuracy of the measurements (80). Nonetheless, a few limitations should be considered. First, the cross-sectional nature of the current study limited the ability to suggest a causal association between meat consumption and inflammation. Second, small errors might exist in the dietary assessment, mainly because of misremembering, overestimation, or underestimation of dietary intake and misclassification errors. Third, since our study included women only, the results are not generalizable to men and even to women with normal weight and other countries. fourth, cooking methods as important confounders didn't assess in the present study. Fifth, meat quality from the conditions of animal husbandry, especially environmental toxins, the type of food and water consumed, environmental conditions, to the stage of the cooking method and process, even genetic variation in dietary response, the culture and food habits of each society for eating of all types of meat can influence the relation of all kinds of meat with inflammation on that people. Sixth, further research must take into account other variables, such as menopause and the participant's hormonal state, which may have an impact on the accuracy of the findings. Seventh, the consumption ratio of all kinds of meats white to red meat or proceed meat, probably affects the inflammation that it is not possible to control the effect by only adjusting it in the analysis stage, and there is a need to design clinical trial studies in this field by proper diet intervention. Finally, although all the analyses were adjusted for potential confounders, residual confounding may still exist.

## Conclusion

In conclusion, according to the results, it seems that the greater consumption of white meat with the mentioned mechanisms is probably negatively related to the inflammatory and metabolic factors. In contrast, the consumption of red and processed meat is positively related to the level of inflammatory factors in overweight and obese women. Of course, a higher intake of red meat had shown an association with a lower level of Gal-3 and insulin resistance, which seems that it is due to the amount of consumption that is lower than the average intake of the other population, and probably with more consumption of red meat, we might have faced different results. Perhaps the situation is clear regarding the recommendation to reduce the consumption of processed meats. However, there is a risk regarding the reduction of red meat consumption despite the reduction of inflammation, and there is concern regarding the consumption of white meat especially sea foods in Iran where some are habitual and some global concerns. Although in this suggestion, we must consider that Iran's estimated anemia prevalence ranges from 10 to 30%, with greater rates among children and teenagers, women with the prominent role of iron deficiency (81), and on the other hand, apart from poultry, seafood consumption in Iran is low due to eating habits and its consumption rate per capita is much lower (82). That change these conditions requires awareness of advantages and the provision of conditions. Other global concerns and risks are heavy metals pollution and the poisoning in sea foods (83, 84). Overall, it seems that it is very difficult to conclude the relationship of meat with inflammatory markers because limitations mentioned. It seems that to make a better judgment and suggestion for public health policy there is still a need for comprehensive studies to investigate the amount of meat consumed compared to the daily dietary protein requirement of individuals, the total energy received from that, along with taking into account controlling cooking methods, eating habits, etc. In addition, long-term controlled feeding studies are needed to confirm the causality of these associations and potential mediating pathways to determine optimal preventative dietary strategies for the progression of inflammation and inflammation-related diseases.

## Data availability statement

The raw data supporting the conclusions of this article will be made available by the authors, without undue reservation.

## Ethics statement

The studies involving human participants were reviewed and approved by Tehran University of Medical Sciences (Ethics Number: IR.TUMS.VCR.REC.1395.1597). The patients/participants provided their written informed consent to participate in this study.

## Author contributions

FSH, DH, and AM wrote the paper. FSH performed the statistical analyses and revised the paper. KHM had full access to all of the data in the study and took responsibility for the integrity and accuracy of the data. AW, RB, and KS revised the paper. All authors read and approved the final manuscript.

## Funding

This study was funded by grants from the Tehran University of Medical Sciences (TUMS) (Grant ID: 95-03-161-33142 and 96-01-161-34479).

## Conflict of interest

The authors declare that the research was conducted in the absence of any commercial or financial relationships that could be construed as a potential conflict of interest.

## Publisher's note

All claims expressed in this article are solely those of the authors and do not necessarily represent those of their affiliated organizations, or those of the publisher, the editors and the reviewers. Any product that may be evaluated in this article, or claim that may be made by its manufacturer, is not guaranteed or endorsed by the publisher.

## Abbreviations

FFQ: food frequency questionnaire
ANOVA: one-way analysis of variance
ANCOVA: analysis of covariance
MCP-1: macrophage inflammatory protein
hs-CRP: high-sensitivity C-reactive protein
PAI-1: plasminogen activator inhibitor 1
HOMA-IR: homeostasis model assessment of insulin resistance
Gal-3: Galectin-3
TGF-b: transforming growth factor
IL-1: interleukin 1
SFA: saturated fatty acid
IL-6: interleukin 6
BMI: body mass index
TUMS: Tehran University of Medical Sciences
FFQ: food frequency questionnaire
WC: waist circumference
TG: triglyceride
HC: hip circumference
NC: neck circumference
WHR: waist-to-hip ratio
WHtR: weight-to-height ratio
SMM: skeletal muscle mass
FFM: fat-free mass
BFM: body fat mass
BMC: bone mass content
SLM: soft lean mass
FMI: fat mass index
FFMI: fat-free mass index
FBS: fasting blood sugar
Chole: total cholesterol
LDL-c: Low-density lipoprotein cholesterol
HDL-c: High-density lipoprotein cholesterol
SGPT: Serum glutamic pyruvic transaminase
SGOT: serum glutamic-oxaloacetic transaminase
SBP: Systolic blood pressure
DBP: diastolic blood pressure
IPAQ: International Physical Activity Questionnaire
MET: Metabolic Equivalents
β: beta
CI: Confidence Interval
CVD: cardiovascular disease
PAH: polycyclic aromatic hydrocarbons
TNF-a: necrosis factor-alpha
GOF: goodness of fit
R2: R squared
AGEs: advanced glycation end products
NO: nitric oxide
eNOS: endothelial nitric oxide synthase
DHN-MA: Mercapturic acid of dihydroxynonane
HNE: hydroxynonenal
MUFA: monounsaturated fatty acids
SFA: saturated fatty acid
EPA: eicosapentaenoic acid
DHA: Docosahexaenoic acid.
